# Regulation of Tissue Growth by the Mammalian Hippo Signaling Pathway

**DOI:** 10.3389/fphys.2017.00942

**Published:** 2017-11-24

**Authors:** Kevin I. Watt, Kieran F. Harvey, Paul Gregorevic

**Affiliations:** ^1^Muscle Research and Therapeutics, Baker Heart and Diabetes Institute, Melbourne, VIC, Australia; ^2^Department of Diabetes, Monash University, Melbourne, VIC, Australia; ^3^Department of Pathology, University of Melbourne, Melbourne, VIC, Australia; ^4^Organogenesis and Cancer Programme, Peter MacCallum Cancer Centre, Melbourne, VIC, Australia; ^5^Sir Peter MacCallum Department of Oncology, University of Melbourne, Melbourne, VIC, Australia; ^6^Department of Anatomy and Developmental Biology, and Biomedicine Discovery Institute, Monash University, Clayton, VIC, Australia; ^7^Department of Physiology, University of Melbourne, Melbourne, VIC, Australia; ^8^Department of Biochemistry and Molecular Biology, Monash University, Clayton, VIC, Australia; ^9^Department of Neurology, University of Washington School of Medicine, Seattle, WA, United States

**Keywords:** hippo signaling pathway, YAP, TAZ, integrative physiology, cell signaling

## Abstract

The integrative control of diverse biological processes such as proliferation, differentiation, apoptosis and metabolism is essential to maintain cellular and tissue homeostasis. Disruption of these underlie the development of many disease states including cancer and diabetes, as well as many of the complications that arise as a consequence of aging. These biological outputs are governed by many cellular signaling networks that function independently, and in concert, to convert changes in hormonal, mechanical and metabolic stimuli into alterations in gene expression. First identified in *Drosophila melanogaster* as a powerful mediator of cell division and apoptosis, the Hippo signaling pathway is a highly conserved regulator of mammalian organ size and functional capacity in both healthy and diseased tissues. Recent studies have implicated the pathway as an effector of diverse physiological cues demonstrating an essential role for the Hippo pathway as an integrative component of cellular homeostasis. In this review, we will: (a) outline the critical signaling elements that constitute the mammalian Hippo pathway, and how they function to regulate Hippo pathway-dependent gene expression and tissue growth, (b) discuss evidence that shows this pathway functions as an effector of diverse physiological stimuli and (c) highlight key questions in this developing field.

## Introduction

The fine control of biological processes such as cell division, terminal differentiation, cell death (apoptosis) and metabolism in response to changes in the external environment is essential for biological organisms to maintain homeostasis and function appropriately (Purvis and Lahav, 2013). These processes are mediated by intracellular signaling networks including the Transforming-Growth Factor beta (TGF-β), Notch, WNT and Insulin-PI3K-mTOR signaling pathways that act in isolation to control the expression of sub-sets of genes, and as an orchestrated network that collectively influences biological phenotypes (Bedinger and Adams, 2015; Bray, 2016; Hata and Chen, 2016; Masuda and Ishitani, 2017). Recent evidence also implicates the relatively less-well-characterized Hippo pathway as a major signaling pathway governing the cellular response to diverse physiological stimuli (Harvey et al., 2013; Meng et al., 2016). The role of the Hippo pathway in Drosophila (Richardson and Portela, 2017), during early mammalian developmental stages (Sasaki, 2017), or in specific tissues such as the heart (He et al., 2017; Zhang and Del Re, 2017), intestine (Gregorieff and Wrana, 2017), and liver (Patel et al., 2017) have recently been described. In this review, we aim to provide the reader with an outline of the critical elements that form the core Hippo signaling pathway in mammals, and to describe the experimental evidence supporting a vital role for this pathway as a major regulator of tissue growth and differentiation in response to three main forms of external regulation: hormonal, mechanical and metabolic stimuli. We will use mammalian nomenclature in general, unless discussing specific results in other organisms.

## The hippo signaling pathway negatively regulates the activity of YAP and TAZ

**Figure 1:**
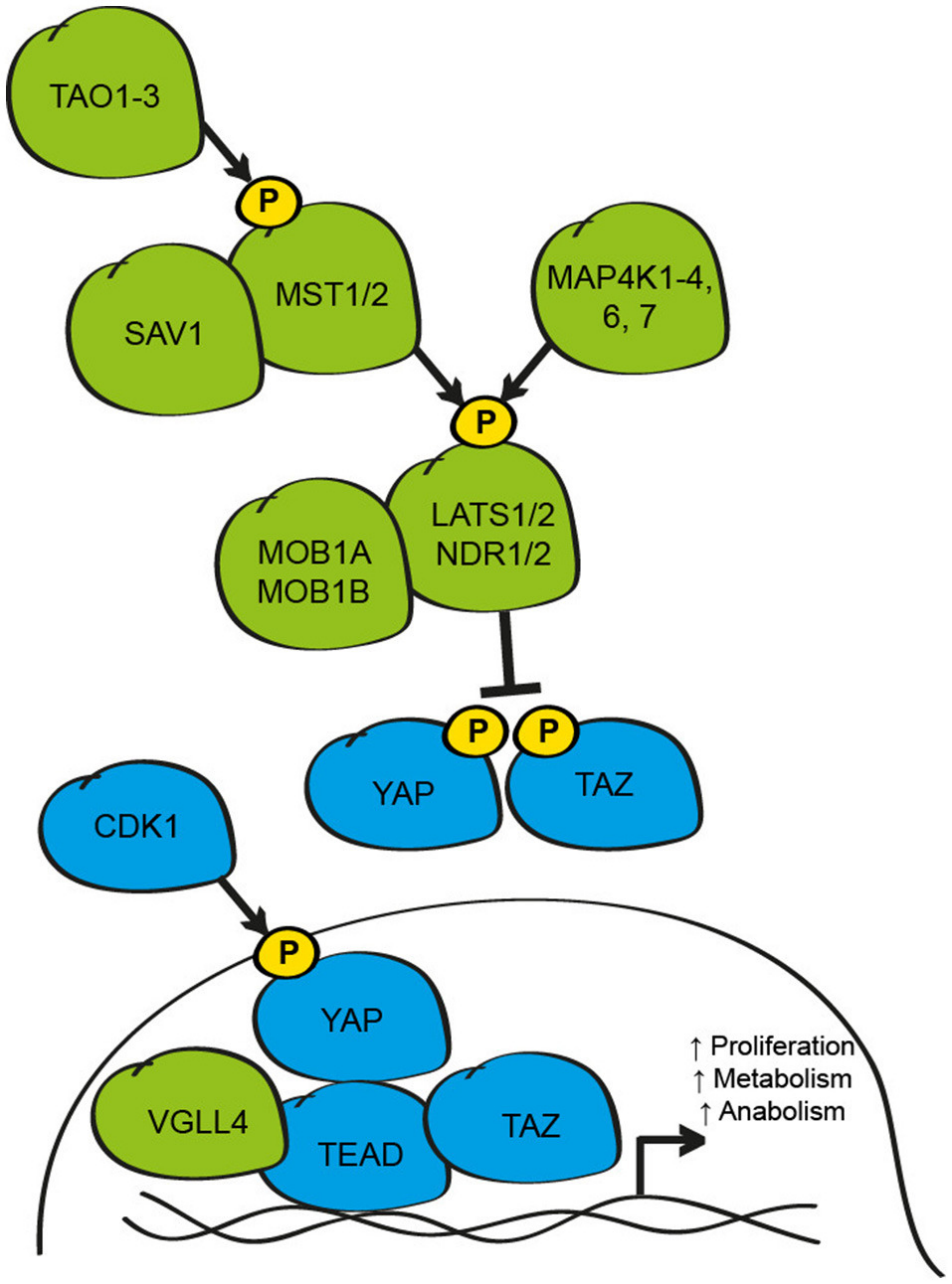
Schematic of the core elements of the mammalian Hippo signaling pathway. The core Hippo pathway kinases (TAO1-3, MST1/2, MAP4K1-4, 6, 7, LATS1/2, and NDR1/2), plus the adaptor proteins (SAV1 and MOB1A/B), function to inhibit the activity of the transcriptional co-activators YAP and TAZ/WWTR1 by phosphorylation at critical serine residues that results in cytoplasmic retention and/or protein degradation. When active, YAP and TAZ bind to TEAD transcription factors to regulate proliferative, metabolic and anabolic gene expression. YAP-TEAD signaling is limited by competitive interaction with VGLL-4. During mitosis, YAP activity can be enhanced by phosphorylation by CDK1. Elements inhibitory to YAP and TAZ activity are shown in green; elements that active/enhance YAP and TAZ activity are shown in blue.

First identified from genetic loss-of-function screens in *Drosophila Melanogaster*, the Hippo signaling pathway has emerged as a powerful regulator of organ size and cell fate across species (Harvey et al., 2013). To date, over 40 proteins have been implicated as members of this diverse pathway; however, the capacity for many of these elements to control Hippo signaling activity is highly context-dependent (Plouffe et al., 2016). Despite this, most stimuli that influence the Hippo pathway converge at a common level to activate, or inhibit, the core Hippo pathway kinases, Mammalian Ste-20 like kinase 1 and 2 (MST1/2) and/or mitogen-activated protein kinase kinase kinase kinases 1-4, 6, and 7 (MAP4K) (Tapon et al., 2002; Harvey et al., 2003; Pantalacci et al., 2003; Udan et al., 2003; Wu et al., 2003; Li et al., 2015; Meng et al., 2015; Zheng et al., 2015; Figure 1). The biochemical regulation of these proteins has been studied mainly with MST1/2, where activation of the kinase is dependent on phosphorylation by the TAO family kinases (TAO1-3) in an activation loop at Thr180/183, respectively (Praskova et al., 2004; Boggiano et al., 2011; Poon et al., 2011). This phosphorylation event is critical for increased catalytic activity and, in the case of MST1/2, the formation of a complex with the adaptor protein WW-domain containing 1 (SAV1) (Pantalacci et al., 2003; Wu et al., 2003; Callus et al., 2006). This interaction appears exclusive to MST1/2 since MAP4K do not interact with SAV1 *in vitro* (Meng et al., 2015; Zheng et al., 2015). Upon activation, MST1/2 or MAP4K can phosphorylate the C-terminal hydrophobic motif of the NDR family kinases Large Tumour Suppressor kinase 1 and 2 (LATS1/2^Thr1079^) and/or the related Nuclear dbf-2 related (NDR) kinases 1/2 (Chan et al., 2005; Hergovich et al., 2009; Li et al., 2015; Meng et al., 2015; Tang et al., 2015; Zheng et al., 2015). Phosphorylated LATS1/2 and/or NDR1/2 then undergo auto-phosphorylation in an activation loop (Chan et al., 2005). MST1/2 also binds the adaptor proteins MOB1A/1B (MOB) leading to phosphorylation on two N-terminal residues; Thr12 and Thr35 (Lai et al., 2005; Praskova et al., 2008). MST1/2 phosphorylation results in activation of MOB and a conformational change that favors binding to LATS1/2 or NDR1/2 kinases (Praskova et al., 2008). When active, the LATS/MOB or NDR/MOB complexes suppress the activity of the transcriptional co-activators Yes-associated protein (YAP1) and transcriptional co-activator with PDZ-binding motif (WWTR1/TAZ) (Huang et al., 2005; Zhao et al., 2007; Lei et al., 2008; Zhang et al., 2015).

YAP and TAZ are the primary effectors of the Hippo pathway in mammals, and are the homologs of the *Drosophila* gene *Yorkie* (Yki) (Huang et al., 2005). While the activity of these proteins can be influenced transcriptionally, YAP and TAZ are mainly regulated by post-translational modifications, in particular by phosphorylation on critical serine residues in a consensus motif (HXRXXS), by LATS1/2 and/or NDR1/2 (Zhao et al., 2007; Lei et al., 2008; Zhang et al., 2015). The importance of this mechanism was highlighted by studies where single and multiple point mutations of critical serine residues (Ser61, 109, 127, 164, and 381; mutated to alanine to create phosphorylation-resistant mutations) were introduced into the human YAP protein (Zhao et al., 2007). Using these mutant YAP proteins, the authors demonstrated that these serine residues are directly phosphorylated by activated LATS1/2 to limit the activity of YAP (Zhao et al., 2007). Similar findings have been reported for TAZ, where LATS phosphorylates Ser66, 89, 117, and 311 (Lei et al., 2008). Of these residues, the best studied are YAP^Ser127^/TAZ^Ser89^ which influence sub-cellular localization and interaction with 14-3-3 binding proteins, and YAP^Ser381^/TAZ^Ser311^ which function as priming sites for a second phosphorylation event at Ser384 by casein kinase 1γ/δ and the subsequent ubiquitination and degradation of YAP or TAZ by the E3 ligase, SCF^β−*TRCP*^ (Zhao et al., 2007, 2010; Liu et al., 2010). While the precise function of the other Serine residues remains unclear, these likely also influence YAP and TAZ activity since mutation of all 5 serine residues results in greater activation of the proteins than mutation of individual residues, at least in cultured cells (Zhao et al., 2009).

Counter to inhibitory phosphorylation by LATS1/2, in mitotically active cells during the G2-M phase of the cell cycle, YAP and TAZ activity is enhanced by phosphorylation by cyclin-dependent kinase 1 (CDK1) at multiple threonine and serine residues; findings that may explain the efficacy of CDK inhibitors under certain circumstances (Yang et al., 2013; Zhao et al., 2014; Pegoraro et al., 2015). Independent of direct inhibition by LATS1/2, the activity of YAP and TAZ can also be influenced by physical retention of the proteins in the cytosol by the Angiomotin family proteins (AMOT, AMOT1L, and AMOT2L) in a LATS1/2 dependent- or independent-manner (Chan et al., 2011; Paramasivam et al., 2011; Wang et al., 2011; Zhao et al., 2011), by disruption of protein-protein interactions necessary for transcriptional activity due to phosphorylation by kinases such as AMP-activated protein kinase (AMPK1) (DeRan et al., 2014; Mo et al., 2015; Wang et al., 2015), interaction with tyrosine kinases including YES and Src (Vassilev et al., 2001), the STRIPAK PP2A phosphatase complex (Ribeiro et al., 2010), methylation by the methyltransferase SET-7 at lysine 494 (Oudhoff et al., 2013), sumolyation by the promyelocytic leukemia protein (PML) (Lapi et al., 2008) and acetylation by the nuclear acetyltransferases CREB binding protein (CBP) and p300 (Hata et al., 2012). Collectively, the co-ordinated influence of these post-translational modifications functions to limit the activity of YAP and/or TAZ.

When Hippo pathway activity is low, YAP and TAZ accumulate predominantly in the nucleus of the cell where they can interact with transcription factors to regulate gene expression including p73, Tbx-5, Smad proteins, FoxO, and Runx (Strano et al., 2001; Ferrigno et al., 2002; Zaidi et al., 2004; Murakami et al., 2005; Varelas et al., 2010b; Rosenbluh et al., 2012; Shao et al., 2014). At this level, Hippo signaling may intersect with a number of other signaling pathways to control subsets of genes during specific cellular contexts (Alarcon et al., 2009; Azzolin et al., 2014). However, the most significant interacting partners of YAP and TAZ are the TEA domain family member transcription factors, (TEAD 1-4; *Scalloped* (Sd) in *Drosophila*) that are essential under most conditions for YAP and TAZ to promote growth (Vassilev et al., 2001; Wu et al., 2008; Zhang et al., 2008; Zhao et al., 2008). The interaction between YAP and TEAD, and TAZ and TEAD is mediated via a C-terminal binding domain in YAP/TAZ and an N-terminal binding domain in TEAD (Vassilev et al., 2001; Zhao et al., 2008). Solving the crystal structure of the YAP-TEAD and TAZ-TEAD complexes has identified the critical regions and residues necessary for this interaction (Chen et al., 2010; Li et al., 2010; Kaan et al., 2017). These studies support previous genetic and bio-chemical studies *in vitro* and *in vivo*, as well as genomic studies of individuals carrying mutations in TEAD1 at Tyr421 who develop Sveinsson's Chorioretinal atrophy, a rare autosomal-dominant disease that leads to degeneration of the photoreceptor cells in the eye (Fossdal et al., 2004).

While the over-expression of YAP or TAZ results in TEAD-dependent gene expression, the over-expression of TEAD or Sd alone leads to the depression of many of these target genes (Koontz et al., 2013). This fascinating observation lead to the demonstration that YAP binding to TEAD results in the de-repression of many of these genes, which under basal conditions are suppressed by TEAD in complex with the Vestigal-like 4 protein (VGLL-4; *Tgi* in *Drosophila*) (Koontz et al., 2013). In these elegant studies, it was demonstrated that under Hippo pathway active conditions, where YAP activity is suppressed, a TEAD/VGLL-4 complex represses the expression of target genes. When the Hippo pathway is inhibited, YAP physically competes with VGLL-4 for TEAD binding, resulting in de-repression of target genes (Koontz et al., 2013). In addition to the activation of YAP and TAZ, CDK1 also phosphorylates VGLL-4 during mitosis to limit binding to TEAD and repression of target genes (Zeng et al., 2017). While Yki/Sd binding occurs mainly in the promoter region of target genes (Oh et al., 2013), active YAP-TEAD and TAZ-TEAD complexes bind mainly to distal enhancer regions where they interact with elements of the transcriptional machinery such as the Mediator complex to influence gene expression (Galli et al., 2015; Kim et al., 2015; Zanconato et al., 2015).

## Control of organ size by YAP and TAZ

The first identified, and best studied, role of the Hippo pathway is the regulation of organ size. This was initially demonstrated during the development of *Drosophila melanogaster* imaginal discs, where loss of upstream elements, or activation of Yki results in a profound over-growth phenotype of epithelial tissues, caused by ectopic cell proliferation, increased progression through the cell cycle and impaired apoptosis (Tapon et al., 2002; Harvey et al., 2003; Pantalacci et al., 2003; Udan et al., 2003; Wu et al., 2003; Huang et al., 2005; Li et al., 2015; Meng et al., 2015; Zheng et al., 2015). Subsequent studies have shown that YAP and TAZ function in a similar manner during the early stages of mammalian embryonic development, and in the specialized development of a number of mammalian tissues, demonstrating the conserved nature of this function for the Hippo pathway (Morin-Kensicki et al., 2006; Camargo et al., 2007; Dong et al., 2007). While the functional output and requirement for TEAD is conserved across species (Hilman and Gat, 2011), the magnitude and complexity of the genes controlled by YAP-TEAD and TAZ-TEAD complexes in mammals differs in tissue- and temporal-specific manners (Meng et al., 2016). Thus, it is likely that YAP and TAZ influence mammalian cell proliferation and apoptosis by controlling the expression of tissue-specific gene signatures, rather than a common subset of target genes. However, recent reports demonstrate in *Drosophila* and a number of mammalian cell types, that Yki/YAP activation is associated with the upregulation of genes encoding Hippo pathway upstream kinases and adaptor proteins, including Neurofibromin 2 (NF2) and LATS2 (Dai et al., 2015; Moroishi et al., 2015; Park G. S. et al., 2016). The induction of these genes forms a negative feedback loop that acts to limit the transcriptional activity of YAP and TAZ, and is likely a critical mechanism to ensure proper development of mammalian tissues indicating that some subsets of target genes are likely common between mammalian tissues (Park G. S. et al., 2016).

In addition to the well-described developmental role for YAP and TAZ, a number of studies now implicate these genes as critical gate-keepers of stem cell expansion and differentiation in adult tissues. The first example of this in mammals was in the mouse intestine where activation of YAP leads to the rapid proliferation of the undifferentiated stem/progenitor cell population (Camargo et al., 2007). Subsequent studies have demonstrated similar findings in the progenitor cells of the skin, intestine, brain and skeletal muscle (Cao et al., 2008; Gee et al., 2011; Schlegelmilch et al., 2011; Beverdam et al., 2013; Tremblay et al., 2014; Sun et al., 2017). Concurrently, appropriate regulation of tissue homeostasis and regeneration requires the subsequent down-regulation of YAP for terminal differentiation to proceed, as demonstrated in genetic mouse models expressing active YAP mutants, or lacking upstream signaling elements (Cao et al., 2008; Lian et al., 2010; Judson et al., 2012; Tremblay et al., 2014; Sun et al., 2017). While YAP and TAZ function in a redundant manner in many settings, this appears more complex during the differentiation of skeletal muscle cells where YAP inhibits, while TAZ promotes terminal differentiation and regeneration (Jeong et al., 2010; Watt et al., 2010; Park G. H. et al., 2014; Mohamed et al., 2016; Sun et al., 2017). These findings have broad implications for regenerative medicine and suggest that appropriate modulation of YAP and/or TAZ activity may be an approach that can be utilized to treat degenerative conditions. However, the efficacy of such an approach is likely to be tissue- and context-dependent, as demonstrated in the mammalian heart where YAP activation can enhance the regenerative capacity of the heart, but only at early stages of post-natal life (Xin et al., 2013). Caution is also warranted with strategies aimed at modulating YAP activity in tissues such as the skin which display profound phenotypes upon activation and inhibition of YAP-TEAD activity in the adult (Schlegelmilch et al., 2011). However, since not all tissue types display phenotypes in response to loss of YAP or TAZ under basal conditions e.g., the intestine or mammary gland (Cai et al., 2010; Chen et al., 2014), targeting this pathway in a tissue-specific manner may still offer great promise for the treatment of a number of conditions.

In addition to the role of the pathway as a regulator of cell division and differentiation, growing evidence supports a role for the Hippo pathway as a mediator of cell size in post-mitotic tissues such as skeletal muscle and the heart, where activation of YAP directly, or by inhibition of LATS2, causes hypertrophy by influencing the rates of protein synthesis, without altering cell number (Matsui et al., 2008; Goodman et al., 2015; Watt et al., 2015). As in epithelial cells, the ability for YAP to regulate basal tissue size in striated muscle appears mostly dependent on TEAD (Watt et al., 2015) providing further evidence for the critical role for the YAP-TEAD and TAZ-TEAD complexes as the primary effectors of Hippo signaling in mammals.

## Integration of hormonal signaling by the hippo pathway

**Figure 2:**
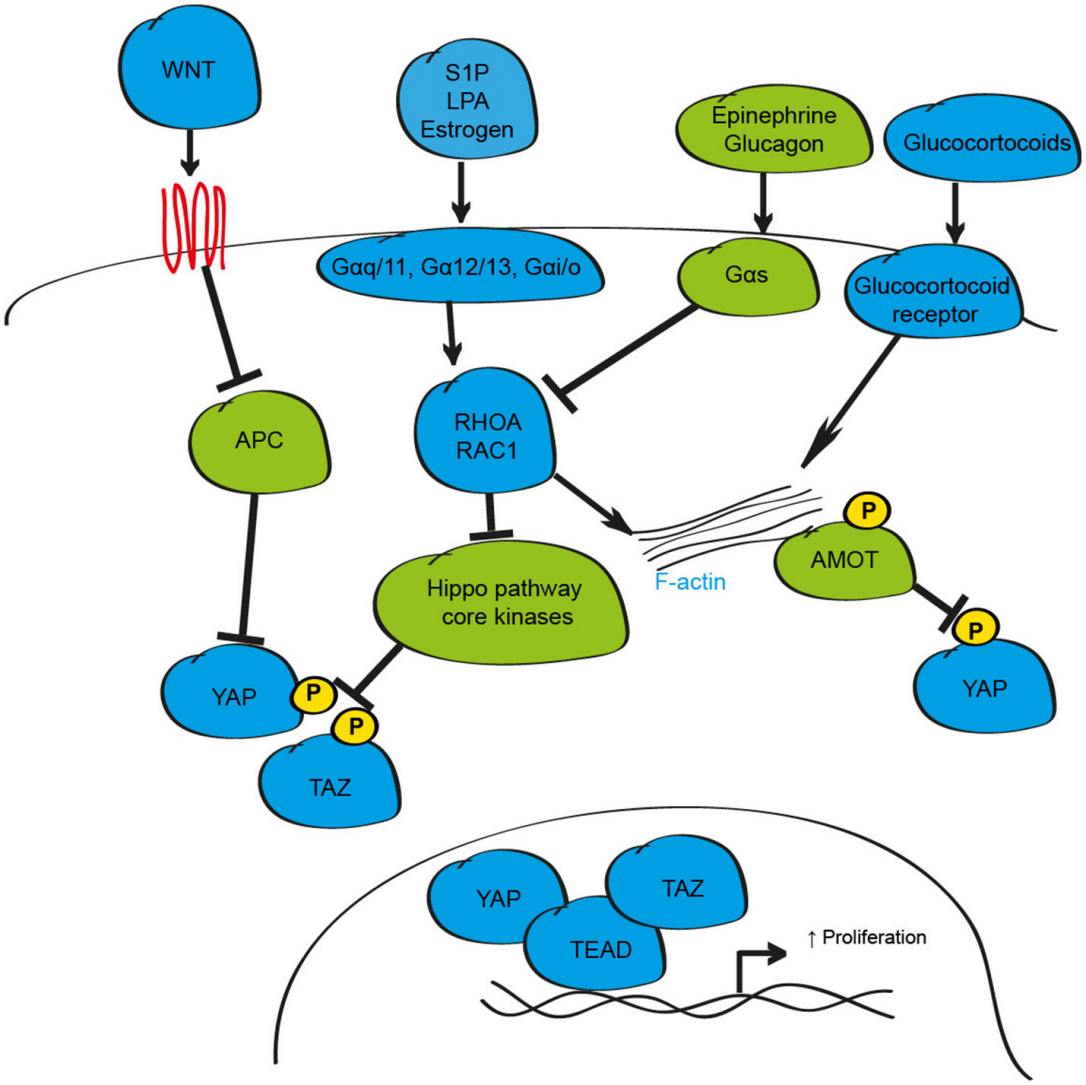
Schematic depicting the hormonal regulation of the mammalian Hippo signaling pathway. In response to hormonal stimuli including S1P, LPA, Estrogen, Epinephrine and Glucagon, the activity of YAP and TAZ can be enhanced via the GPCR G-proteins Gαq/11, Gα12/13, or Gαi/o, or inhibited via Gαs signaling axis. When active, these stimuli influence YAP and TAZ phosphorylation/localization through modulation of F-actin/RHOA/RAC1. YAP can also be phosphorylated and sequestered by the AMOT family of proteins. In addition to regulation by GPCR signaling, YAP, and TAZ activity is altered by secreted proteins such as WNT and by glucocorticoids.

One common approach that cells utilize to modulate intra-cellular responses to changes in the external environment is the synthesis and release of soluble factors such as hormones, growth factors and diffusible molecules including insulin and glucose (Bedinger and Adams, 2015). While genetic studies were instrumental for elucidating the critical intracellular signaling proteins that regulate YAP and TAZ activity, defining how the Hippo pathway is activated by external stimuli to respond to changes in the environment has proved more elusive. Studies exploring this area demonstrated that soluble factors including lysophosphatidic acid (LPA), Sphingosine-1-phosphate (S1P), epinephrine, estrogen, and glucagon can activate, or inhibit, YAP and TAZ via the Hippo pathway by their cognate G-protein coupled receptors (Miller et al., 2012; Yu et al., 2012; Zhou et al., 2015; Figure 2). When activated by such soluble factors, these receptors recruit and couple with their associated G-protein sub-units to engage the small GTPases RHOA and RAC1 leading to alterations in LATS1/2 activity (Yu et al., 2012; Plouffe et al., 2016). In most cases, stimulation of Gα_q/11_, Gα_12/13_, and Gα_i/o_ subunits is reported to activate YAP and TAZ, while Gα_s_ subunits inhibit; however, this is likely dependent on many factors including receptor recycling rate, G-subunit protein levels, and expression of downstream signaling effector proteins meaning that individual sub-units could both activate, or inhibit, YAP and TAZ depending on the cellular context (Yu et al., 2012; Plouffe et al., 2016). Of note, activating mutations in G protein sub-units are observed in a number of cancer sub-types that display increased YAP protein (Yu et al., 2012; Feng et al., 2014; Zhou et al., 2015).

A number of soluble signaling proteins that act independently of the G-protein coupled receptor signaling pathways have also been proposed to influence cell proliferation, differentiation and inflammation through YAP and TAZ including Glucocorticoids, Epidermal Growth Factor Receptor (EGFR), Insulin-like growth factor-1, WNT, TGF-β, Bone Morphogenic Proteins (BMPs) and the cytokines leukemia inhibitory factor (LIF) and Interlukin-6 (IL6) (Alarcon et al., 2009; Tamm et al., 2011; Strassburger et al., 2012; Reddy and Irvine, 2013; Azzolin et al., 2014; Haskins et al., 2014; Park H. W. et al., 2015; Taniguchi et al., 2015; Sorrentino et al., 2017). The importance of these interactions with Hippo signaling in many cell and tissue types, especially during non-pathological conditions, has yet to be demonstrated. However, a growing body of evidence in multiple tissues supports the suggestion that interactions at multiple levels with the WNT signaling pathway may influence YAP and TAZ activity to alter tissue growth in multiple settings. The interaction between these pathways is complex and likely context-dependent, but can include transcriptional regulation of YAP expression, effects on YAP and TAZ protein stability and localization, and modulation of Hippo pathway target gene expression (Varelas et al., 2010a; Heallen et al., 2011; Azzolin et al., 2012, 2014; Rosenbluh et al., 2012). In addition to WNT-mediated control of Hippo signaling, YAP-TEAD, and TAZ-TEAD may also influence WNT activity through transcriptional regulation of WNT signaling pathway elements (Heallen et al., 2011).

## Hippo signaling and mechano-transduction

**Figure 3:**
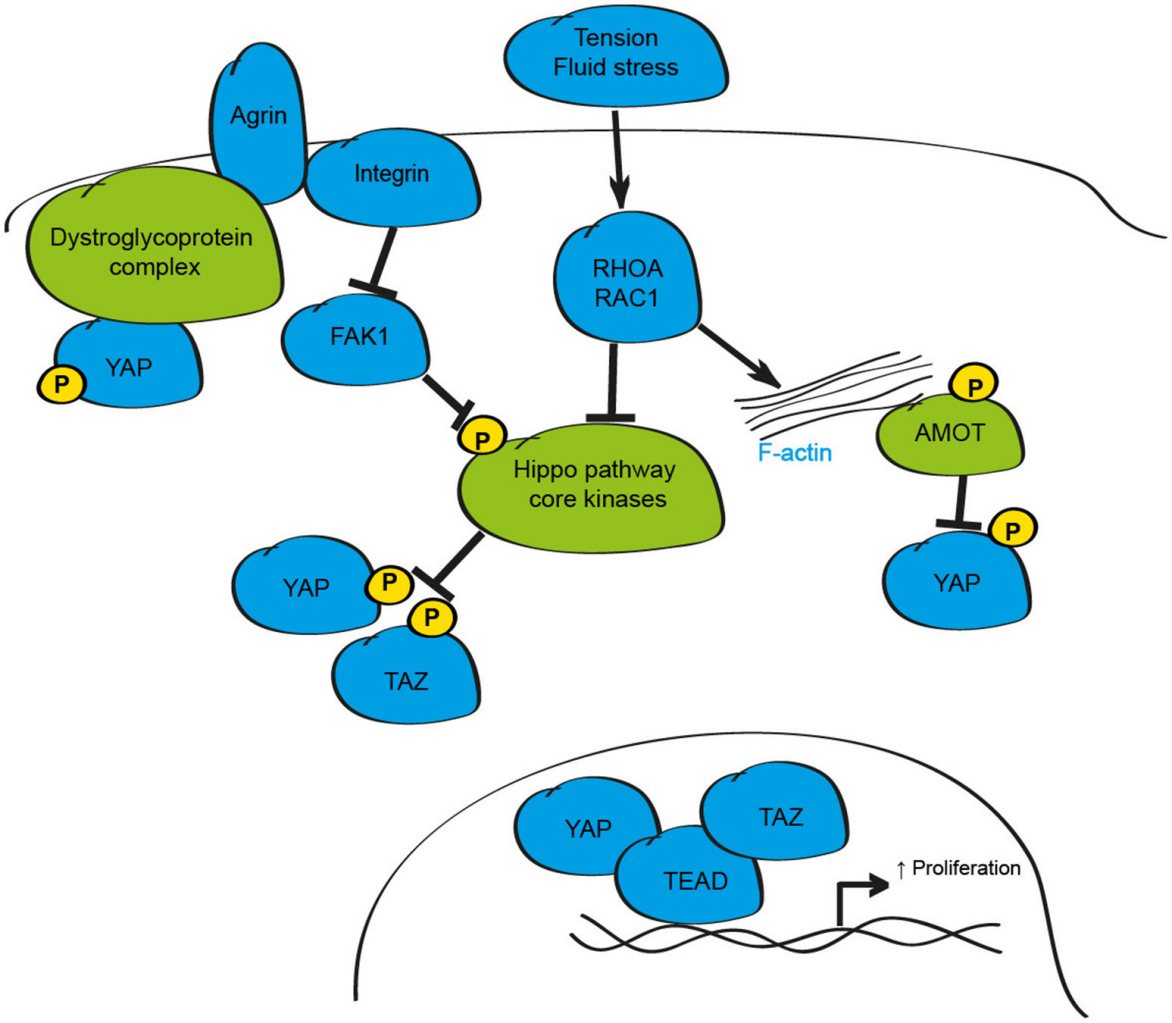
Schematic depicting the mechanical regulation of the mammalian Hippo signaling pathway. Changes in mechanical tension, shear stress, and cell-cell contact can alter YAP and TAZ activity through the F-actin/RHOA/RAC1 axis. Additionally, the proteoglycan Agrin modulates YAP and TAZ activity via Integrin/FAK-mediated activation of the Hippo core kinases and by modulation of the dystroglyoprotein complex.

In addition to soluble molecules, regulation of physiological function in the local microenvironment is highly dependent on changes in mechanical properties derived from alterations in cell shape, fluid shear-stress or cell-cell contact (Finch-Edmondson and Sudol, 2016). Recent experimental evidence highlights a role for YAP and TAZ as critical effectors of mechanical signals (Figure 3). The first findings linking changes in physical dynamics to Hippo signaling came from studies showing that as cells divide and make contact with neighboring cells, YAP phosphorylation increased and was re-localized to the cytosol of the cell (Zhao et al., 2007). Growing evidence supports the conclusion that in addition, and in isolation of cell-cell contact, changes in mechanical properties of the ECM, cell geometry and polymerisation of the F-actin cytoskeleton are essential inputs that influence the activity of YAP and TAZ (Dupont et al., 2011; Wada et al., 2011; Aragona et al., 2013; Bertrand et al., 2014).

When grown in isolation on stiff substrates, or under conditions where cells can spread, YAP and TAZ are active and localize to the nucleus (Dupont et al., 2011; Wada et al., 2011; Aragona et al., 2013). However, in soft matrices, or when cultured in small surface areas so that they are compressed, YAP and TAZ are inhibited and re-localize to the cytosol (Dupont et al., 2011; Aragona et al., 2013). These effects were proposed to be mediated by Hippo pathway dependent- and independent-mechanisms; however, as in the case of soluble factors, redundancy with kinases such as NDR1/2 was not tested, and so the precise mechanism of action remains unclear. Despite this, studies assessing the mechanisms downstream of mechanical cues collectively demonstrate that mechanical stimuli influence YAP and TAZ activity through alterations in F-actin polymerisation, and by changes in the activity of the small GTPases RHOA and RAC1 (Dupont et al., 2011; Fernandez et al., 2011; Sansores-Garcia et al., 2011; Aragona et al., 2013; Plouffe et al., 2016). Disruption of the F-actin-RHOA/RAC1-YAP/TAZ pathway has been implicated in the specification of cell identify during development, in the activation and differentiation of various cells and in the maintenance of vertebrate three-dimensional tissue structure and tension, as well as in response to fluid flow stresses to influence the stability of blood vessels during development and the formation atherosclerotic plaques in APOE^−/−^ mice, demonstrating the significance of this mode of regulation to a number of cellular processes and disease-relevant settings (Sansores-Garcia et al., 2011; Calvo et al., 2013; Liu et al., 2015; Porazinski et al., 2015; Wang K. C. et al., 2016; Wang L. et al., 2016; Nakajima et al., 2017; Totaro et al., 2017).

The critical pathways that are required to relay external mechanical forces in the extracellular matrix to influence Hippo signaling in mammalian tissues have only recently been identified. Studies in liver and heart cells suggest that Agrin, a proteoglycan that regulates formation and maintenance of the neuromuscular junction, and the dystroglycoprotein-complex (DGC), a multiprotein complex that links the extra-cellular matrix and actin cytoskeleton, may both influence Hippo signaling in mammalian tissues (Bassat et al., 2017; Chakraborty et al., 2017; Morikawa et al., 2017). While Agrin, via Integrin/PAK1 signaling, regulates YAP activity through the core Hippo kinases (Chakraborty et al., 2017), the DGC appears to limit YAP activity by physical interaction of phosphorylated YAP through the DGC component Dag1 (Morikawa et al., 2017). Recent evidence in cardiomyocytes suggests that Agrin-dependent activation of YAP is also mediated by disruption of the DGC complex in settings of cardiomyocyte regeneration providing further evidence for this axis as a regulator of YAP activity (Bassat et al., 2017). Given the critical role of Agrin-Integrin and DGC protein complexes during development, and in neurodegenerative and neuromuscular diseases, further study of these mechanistic links may offer exciting strategies that can be exploited to modulate YAP and TAZ activity in a range of disease states.

## Cellular metabolism and the hippo pathway

**Figure 4:**
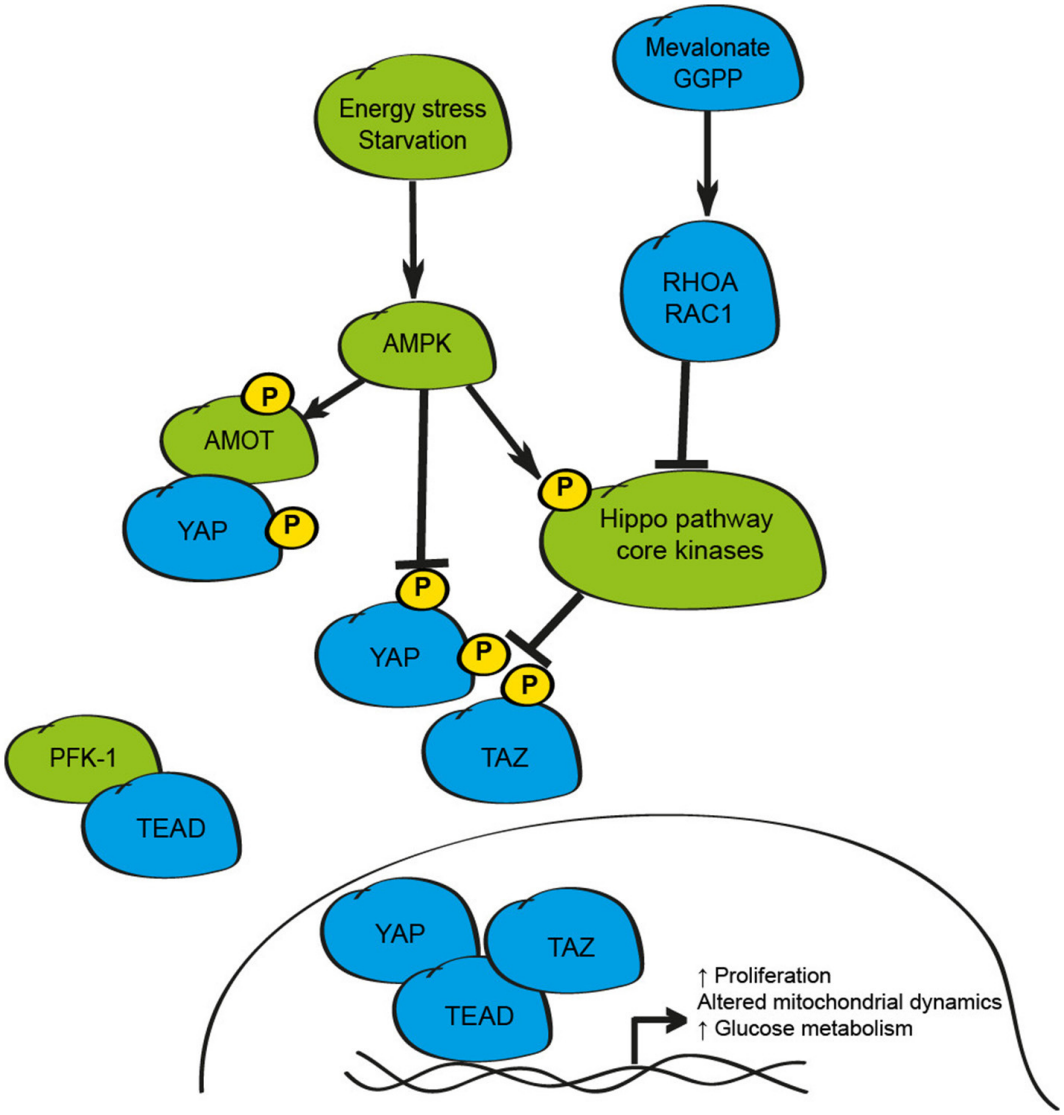
Schematic depicting the metabolic regulation of the mammalian Hippo signaling pathway. Energy stress as observed in settings of glucose withdrawal/starvation inhibits YAP activity via activation of AMPK leading to phosphorylation of AMOT, YAP, or disruption of the YAP/TEAD complex. Metabolic pathways including the mevalonate/HMD CoA reductase-GGPP pathway also influence YAP and TAZ activity via RHOA/RAC1. Activity of this pathway can also be limited by disruption of YAP-TEAD binding by PFK-1.

Integration of tissue-specific, and whole-body, metabolic signaling responses is essential to ensure homeostasis under basal conditions. Furthermore, the dysregulation of metabolic pathways in many tissues from lipid metabolism toward anaerobic glycolysis (the Warburg effect) is an established event in the development and progression of many cancers, and in the activation of stem cell populations (Agathocleous and Harris, 2013; Zhou et al., 2017). Recent studies highlight a number of essential metabolic pathways that appear to function via modulation of YAP and TAZ (Figure 4).

The first link between the Hippo pathway and cellular metabolism was the demonstration that the inhibition of the mevalonate/HMG-CoA reductase pathway, a critical mediator of cholesterol and isoprenoids production, was sufficient to promote cytoplasmic retention, and to impair the transcriptional activity of YAP and TAZ in breast cancer cells (Sorrentino et al., 2014; Wang et al., 2014). The mevalonate pathway acts to enhance YAP and TAZ activity via geranylgeranyl pyrophosphate (GGPP) and RHOA suggesting that the small GTPases RHOA and RAC1 may form common intercellular mediators of YAP/TAZ function in response to metabolic, hormonal and mechanical stimuli (Sorrentino et al., 2014; Wang et al., 2014). The significance of these findings in non-cancerous cell types is less clear; yet inhibition of HMG-CoA reductase by statin exposure in developing mouse embryos prevents blastocyst formation and impairs YAP activity highlighting a role for this axis during early mammalian development (Alarcon and Marikawa, 2016).

The activity of YAP and TAZ can also be impacted by the cellular concentrations of glucose and glucose-responsive pathways (Adler et al., 2013; DeRan et al., 2014; Enzo et al., 2015; Mo et al., 2015; Wang et al., 2015). Energy stress, as induced by culturing cells in glucose-free conditions, results in inhibition of YAP activity in mouse hepatocytes *in vivo* as demonstrated by starvation/re-feeding experiments (Wang et al., 2015). While these studies collectively demonstrate that glucose withdrawal inhibits YAP activity, the underlying mechanisms reported differ between studies and include AMPK1-mediated phosphorylation of AMOTL1, direct phosphorylation of YAP on multiple serine residues by AMPK1, disruption of the YAP-TEAD complex by AMPK1 phosphorylation at Ser94, and physical interaction between TEADs and phosphofructokinase-1 (PFK-1), a rate-limiting enzyme in the glycolysis pathway (Adler et al., 2013; DeRan et al., 2014; Enzo et al., 2015; Mo et al., 2015; Wang et al., 2015).

In addition to being responsive to changes in cellular metabolism, the Hippo pathway appears to control key metabolic components to influence cell proliferation under certain conditions including transcriptional control of glucose transporters, gluconeogenic gene expression, amino acid transporters, and elements of the glutamine metabolism pathways (Hansen et al., 2015; Wang et al., 2015; Cox et al., 2016; Park Y. Y. et al., 2016; Hu et al., 2017). Further to the effect of these metabolism-induced changes on cell division, the manipulation of YAP and TAZ can also influence mitochondrial dynamics, through enhancing the rates of mitochondrial fusion and fission, by transcriptional up-regulation of genes such as *Opa-1* and *Mfn2*, critical elements controlling these vital cellular processes (Nagaraj et al., 2012; von Eyss et al., 2015). These findings highlight the interplay between Hippo signaling and multiple metabolic processes and demonstrate further mechanisms of growth control by this pathway that could be exploited in settings such as cancer, metabolic disease or regenerative medicine.

## Concluding remarks

Here, we summarize evidence supporting an essential role for the Hippo signaling pathway as a critical element integrating hormonal, mechanical and metabolic stimuli during the growth and differentiation of mammalian tissues during development, in the post-natal environment, and in the progression of specific disease states. Together, these findings suggest that targeting the Hippo pathway may be beneficial in particular clinical settings, with inhibition of the pro-proliferative/anti-apoptotic function of YAP and TAZ during the growth of cancer cells of particular interest. Potential approaches that could be used to achieve this goal include inhibitors of the mevalonate pathway (Statins, Bisphosphonates and Geranylgeranyl-transferase inhibitors), RHO GTPases (ROCK inhibitors), or small molecules that disrupt the interaction between YAP and TEAD, such as Verteporfin; many of which are used in clinical practice for other conditions (Dupont et al., 2011; Liu-Chittenden et al., 2012; Sorrentino et al., 2014; Wang et al., 2014). However, studies using these agents have typically limited their analyses to a single cell/tissue type and so the consequences on whole-body physiology are not clear. A greater understanding of this will be critical for the successful development of therapeutics targeting YAP and TAZ; particularly in light of the detrimental effects of inhibiting basal levels of YAP activity in tissues such as skeletal muscle and skin (Schlegelmilch et al., 2011; Watt et al., 2015). Targeting elements of the Hippo pathway using lower effective doses of agents, but as part of a combinatorial therapeutic approach, may therefore be an alternative means to inhibit the Hippo pathway while limiting potential detrimental consequences on other tissues. Such approaches appear to be effective in a number of cell types (Lin et al., 2015; Li et al., 2016; Zhao et al., 2017).

A major limitation of studies investigating the function of the Hippo pathway is the use of genetic manipulation whereby pathway members are exogenously over-expressed or completely deleted from a target tissue; yet complete deletion of pathway members in healthy or diseased tissues is not commonly observed (the exception being the *NF2* gene in select cancers; Harvey et al., 2013). Consequently, assessing the function of Hippo pathway members in specific tissues at physiological and appropriate patho-physiological levels using more elegant approaches such as genome editing with CRISPR/CAS9, where the introduction of point-mutations, protein domain deletions, and activation of genes at the endogenous locus is possible may reveal more relevant information about the role of this pathway in the progression of disease pathologies.

While the significance of the Hippo pathway in many aspects of mammalian biology is becoming increasingly clear, evidence of a role for the Hippo pathway in cellular processes such as the response to hypoxia, autophagy and the unfolded protein response is limited to various tumor cell types, tissue culture models, or specific tissues (Liang et al., 2014; Yan et al., 2014; Wu et al., 2015). Future work will be required to explore how significant alterations in the activity of the Hippo pathway are to these critical cellular functions in non-cancerous tissues if we are to understand the significance of the Hippo pathway in these processes.

In summary, the published literature to date implicates the Hippo signaling pathway as a mediator of tissue growth and function in response to diverse physiological cues. While our understanding of the requirement for the Hippo pathway in mammals has expanded in recent years, the consequence of altered Hippo signaling in specific tissues on whole-body physiology, and the effect of such changes upon disease progression remains unexplored. Future studies aimed at understanding the integrative nature of the Hippo pathway on human physiology will be required to reveal the extent that this pathway influences biological function and the implications of targeting this pathway for clinical benefit.

## Author contributions

KW, KH, and PG wrote and edited the manuscript.

### Conflict of interest statement

The handling Editor declared a shared affiliation, though no other collaboration, with the authors KW and PG. The other authors declare that the research was conducted in the absence of any commercial or financial relationships that could be construed as a potential conflict of interest.

## References

[B1] Adler J. J. Johnson D. E. Heller B. L. Bringman L. R. Ranahan W. P. Conwell M. D.. (2013). Serum deprivation inhibits the transcriptional co-activator YAP and cell growth via phosphorylation of the 130-kDa isoform of Angiomotin by the LATS1/2 protein kinases. Proc. Natl. Acad. Sci. U.S.A. 110, 17368–17373. 10.1073/pnas.1308236110 24101513 PMC3808603

[B2] Agathocleous M. Harris W. A. (2013). Metabolism in physiological cell proliferation and differentiation. Trends Cell Biol. 23, 484–492. 10.1016/j.tcb.2013.05.004 23756093

[B3] Alarcon C. Zaromytidou A. I. Xi Q. Gao S. Yu J. Fujisawa S.. (2009). Nuclear CDKs drive Smad transcriptional activation and turnover in BMP and TGF-beta pathways. Cell 139, 757–769. 10.1016/j.cell.2009.09.035 19914168 PMC2818353

[B4] Alarcon V. B. Marikawa Y. (2016). Statins inhibit blastocyst formation by preventing geranylgeranylation. Mol. Hum. Reprod. 22, 350–363. 10.1093/molehr/gaw011 26908642 PMC4847613

[B5] Aragona M. Panciera T. Manfrin A. Giulitti S. Michielin F. Elvassore N.. (2013). A mechanical checkpoint controls multicellular growth through YAP/TAZ regulation by actin-processing factors. Cell 154, 1047–1059. 10.1016/j.cell.2013.07.042 23954413

[B6] Azzolin L. Panciera T. Soligo S. Enzo E. Bicciato S. Dupont S.. (2014). YAP/TAZ incorporation in the β-catenin destruction complex orchestrates the Wnt response. Cell 158, 157–170. 10.1016/j.cell.2014.06.013 24976009

[B7] Azzolin L. Zanconato F. Bresolin S. Forcato M. Basso G. Bicciato S.. (2012). Role of TAZ as mediator of Wnt signaling. Cell 151, 1443–1456. 10.1016/j.cell.2012.11.027 23245942

[B8] Bassat E. Mutlak Y. E. Genzelinakh A. Shadrin I. Y. Baruch Umansky K. Yifa O.. (2017). The extracellular matrix protein agrin promotes heart regeneration in mice. Nature 547, 179–184. 10.1038/nature22978 28581497 PMC5769930

[B9] Bedinger D. H. Adams S. H. (2015). Metabolic, anabolic, and mitogenic insulin responses: a tissue-specific perspective for insulin receptor activators. Mol. Cell. Endocrinol. 415, 143–156. 10.1016/j.mce.2015.08.013 26277398

[B10] Bertrand A. T. Ziaei S. Ehret C. Duchemin H. Mamchaoui K. Bigot A.. (2014). Cellular microenvironments reveal defective mechanosensing responses and elevated YAP signaling in LMNA-mutated muscle precursors. J. Cell Sci. 127(Pt 13), 2873–2884. 10.1242/jcs.144907 24806962

[B11] Beverdam A. Claxton C. Zhang X. James G. Harvey K. F. Key B. (2013). Yap controls stem/progenitor cell proliferation in the mouse postnatal epidermis. J. Invest. Dermatol. 133, 1497–1505. 10.1038/jid.2012.430 23190885

[B12] Boggiano J. C. Vanderzalm P. J. Fehon R. G. (2011). Tao-1 phosphorylates Hippo/MST kinases to regulate the Hippo-Salvador-Warts tumor suppressor pathway. Dev. Cell 21, 888–895. 10.1016/j.devcel.2011.08.028 22075147 PMC3217187

[B13] Bray S. J. (2016). Notch signalling in context. Nat. Rev. Mol. Cell Biol. 17, 722–735. 10.1038/nrm.2016.94 27507209

[B14] Cai J. Zhang N. Zheng Y. de Wilde R. F. Maitra A. Pan D. (2010). The Hippo signaling pathway restricts the oncogenic potential of an intestinal regeneration program. Genes Dev. 24, 2383–2388. 10.1101/gad.1978810 21041407 PMC2964748

[B15] Callus B. A. Verhagen A. M. Vaux D. L. (2006). Association of mammalian sterile twenty kinases, Mst1 and Mst2, with hSalvador via C-terminal coiled-coil domains, leads to its stabilization and phosphorylation. FEBS J. 273, 4264–4276. 10.1111/j.1742-4658.2006.05427.x 16930133

[B16] Calvo F. Ege N. Grande-Garcia A. Hooper S. Jenkins R. P. Chaudhry S. I.. (2013). Mechanotransduction and YAP-dependent matrix remodelling is required for the generation and maintenance of cancer-associated fibroblasts. Nat. Cell Biol. 15, 637–646. 10.1038/ncb2756 23708000 PMC3836234

[B17] Camargo F. D. Gokhale S. Johnnidis J. B. Fu D. Bell G. W. Jaenisch R.. (2007). YAP1 increases organ size and expands undifferentiated progenitor cells. Curr. Biol. 17, 2054–2060. 10.1016/j.cub.2007.10.039 17980593

[B18] Cao X. Pfaff S. L. Gage F. H. (2008). YAP regulates neural progenitor cell number via the TEA domain transcription factor. Genes Dev. 22, 3320–3334. 10.1101/gad.1726608 19015275 PMC2600760

[B19] Chakraborty S. Njah K. Pobbati A. V. Lim Y. B. Raju A. Lakshmanan M.. (2017). Agrin as a mechanotransduction signal regulating YAP through the Hippo pathway. Cell Rep. 18, 2464–2479. 10.1016/j.celrep.2017.02.041 28273460

[B20] Chan E. H. Nousiainen M. Chalamalasetty R. B. Schafer A. Nigg E. A. Sillje H. H. (2005). The Ste20-like kinase Mst2 activates the human large tumor suppressor kinase Lats1. Oncogene 24, 2076–2086. 10.1038/sj.onc.1208445 15688006

[B21] Chan S. W. Lim C. J. Chong Y. F. Pobbati A. V. Huang C. Hong W. (2011). Hippo pathway-independent restriction of TAZ and YAP by angiomotin. J. Biol. Chem. 286, 7018–7026. 10.1074/jbc.C110.212621 21224387 PMC3044958

[B22] Chen L. Chan S. W. Zhang X. Walsh M. Lim C. J. Hong W.. (2010). Structural basis of YAP recognition by TEAD4 in the hippo pathway. Genes Dev. 24, 290–300. 10.1101/gad.1865310 20123908 PMC2811830

[B23] Chen Q. Zhang N. Gray R. S. Li H. Ewald A. J. Zahnow C. A.. (2014). A temporal requirement for Hippo signaling in mammary gland differentiation, growth, and tumorigenesis. Genes Dev. 28, 432–437. 10.1101/gad.233676.113 24589775 PMC3950341

[B24] Cox A. G. Hwang K. L. Brown K. K. Evason K. Beltz S. Tsomides A.. (2016). Yap reprograms glutamine metabolism to increase nucleotide biosynthesis and enable liver growth. Nat. Cell Biol. 18, 886–896. 10.1038/ncb3389 27428308 PMC4990146

[B25] Dai X. Liu H. Shen S. Guo X. Yan H. Ji X.. (2015). YAP activates the Hippo pathway in a negative feedback loop. Cell Res. 25, 1175–1178. 10.1038/cr.2015.101 26315483 PMC4650621

[B26] DeRan M. Yang J. Shen C. H. Peters E. C. Fitamant J. Chan P.. (2014). Energy stress regulates hippo-YAP signaling involving AMPK-mediated regulation of angiomotin-like 1 protein. Cell Rep. 9, 495–503. 10.1016/j.celrep.2014.09.036 25373897 PMC4223634

[B27] Dong J. Feldmann G. Huang J. Wu S. Zhang N. Comerford S. A.. (2007). Elucidation of a universal size-control mechanism in Drosophila and mammals. Cell 130, 1120–1133. 10.1016/j.cell.2007.07.019 17889654 PMC2666353

[B28] Dupont S. Morsut L. Aragona M. Enzo E. Giulitti S. Cordenonsi M.. (2011). Role of YAP/TAZ in mechanotransduction. Nature 474, 179–183. 10.1038/nature10137 21654799

[B29] Enzo E. Santinon G. Pocaterra A. Aragona M. Bresolin S. Forcato M.. (2015). Aerobic glycolysis tunes YAP/TAZ transcriptional activity. EMBO J. 34, 1349–1370. 10.15252/embj.201490379 25796446 PMC4491996

[B30] Feng X. Degese M. S. Iglesias-Bartolome R. Vaque J. P. Molinolo A. A. Rodrigues M.. (2014). Hippo-independent activation of YAP by the GNAQ uveal melanoma oncogene through a trio-regulated rho GTPase signaling circuitry. Cancer Cell 25, 831–845. 10.1016/j.ccr.2014.04.016 24882515 PMC4074519

[B31] Fernandez B. G. Gaspar P. Bras-Pereira C. Jezowska B. Rebelo S. R. Janody F. (2011). Actin-Capping Protein and the Hippo pathway regulate F-actin and tissue growth in Drosophila. Development 138, 2337–2346. 10.1242/dev.063545 21525075

[B32] Ferrigno O. Lallemand F. Verrecchia F. L'Hoste S. Camonis J. Atfi A.. (2002). Yes-associated protein (YAP65) interacts with Smad7 and potentiates its inhibitory activity against TGF-β/Smad signaling. Oncogene 21, 4879–4884. 10.1038/sj.onc.1205623 12118366

[B33] Finch-Edmondson M. Sudol M. (2016). Framework to function: mechanosensitive regulators of gene transcription. Cell. Mol. Biol. Lett. 21:28. 10.1186/s11658-016-0028-7 28536630 PMC5415767

[B34] Fossdal R. Jonasson F. Kristjansdottir G. T. Kong A. Stefansson H. Gosh S.. (2004). A novel TEAD1 mutation is the causative allele in Sveinsson's chorioretinal atrophy (helicoid peripapillary chorioretinal degeneration). Hum. Mol. Genet. 13, 975–981. 10.1093/hmg/ddh106 15016762

[B35] Galli G. G. Carrara M. Yuan W. C. Valdes-Quezada C. Gurung B. Pepe-Mooney B.. (2015). YAP drives growth by controlling transcriptional pause release from dynamic enhancers. Mol. Cell 60, 328–337. 10.1016/j.molcel.2015.09.001 26439301 PMC4624327

[B36] Gee S. T. Milgram S. L. Kramer K. L. Conlon F. L. Moody S. A. (2011). Yes-associated protein 65 (YAP) expands neural progenitors and regulates Pax3 expression in the neural plate border zone. PLoS ONE 6:e20309. 10.1371/journal.pone.0020309 21687713 PMC3110623

[B37] Goodman C. A. Dietz J. M. Jacobs B. L. McNally R. M. You J. S. Hornberger T. A. (2015). Yes-Associated Protein is up-regulated by mechanical overload and is sufficient to induce skeletal muscle hypertrophy. FEBS Lett. 589, 1491–1497. 10.1016/j.febslet.2015.04.047 25959868 PMC4442043

[B38] Gregorieff A. Wrana J. L. (2017). Hippo signalling in intestinal regeneration and cancer. Curr. Opin. Cell Biol. 48, 17–25. 10.1016/j.ceb.2017.04.005 28527754

[B39] Hansen C. G. Ng Y. L. Lam W. L. Plouffe S. W. Guan K. L. (2015). The Hippo pathway effectors YAP and TAZ promote cell growth by modulating amino acid signaling to mTORC1. Cell Res. 25, 1299–1313. 10.1038/cr.2015.140 26611634 PMC4670996

[B40] Harvey K. F. Pfleger C. M. Hariharan I. K. (2003). The Drosophila Mst ortholog, hippo, restricts growth and cell proliferation and promotes apoptosis. Cell 114, 457–467. 10.1016/S0092-8674(03)00557-9 12941274

[B41] Harvey K. F. Zhang X. Thomas D. M. (2013). The Hippo pathway and human cancer. Nat. Rev. Cancer 13, 246–257. 10.1038/nrc3458 23467301

[B42] Haskins J. W. Nguyen D. X. Stern D. F. (2014). Neuregulin 1-activated ERBB4 interacts with YAP to induce Hippo pathway target genes and promote cell migration. Sci. Signal. 7:ra116. 10.1126/scisignal.2005770 25492965 PMC4648367

[B43] Hata A. Chen Y.-G. (2016). TGF-β signaling from receptors to smads. Cold Spring Harb. Perspect. Biol. 8:a022061. 10.1101/cshperspect.a022061 27449815 PMC5008074

[B44] Hata S. Hirayama J. Kajiho H. Nakagawa K. Hata Y. Katada T.. (2012). A novel acetylation cycle of transcription co-activator Yes-associated protein that is downstream of Hippo pathway is triggered in response to SN2 alkylating agents. J. Biol. Chem. 287, 22089–22098. 10.1074/jbc.M111.334714 22544757 PMC3381167

[B45] He J. Bao Q. Yan M. Liang J. Zhu Y. Wang C.. (2017). The role of Hippo/yes-associated protein signalling in vascular remodelling associated with cardiovascular disease. Br. J. Pharmacol. [Epub ahead of print]. 10.1111/bph.13806 28369744 PMC5866970

[B46] Heallen T. Zhang M. Wang J. Bonilla-Claudio M. Klysik E. Johnson R. L.. (2011). Hippo pathway inhibits Wnt signaling to restrain cardiomyocyte proliferation and heart size. Science 332, 458–461. 10.1126/science.1199010 21512031 PMC3133743

[B47] Hergovich A. Kohler R. S. Schmitz D. Vichalkovski A. Cornils H. Hemmings B. A. (2009). The MST1 and hMOB1 tumor suppressors control human centrosome duplication by regulating NDR kinase phosphorylation. Curr. Biol. 19, 1692–1702. 10.1016/j.cub.2009.09.020 19836237

[B48] Hilman D. Gat U. (2011). The evolutionary history of YAP and the hippo/YAP pathway. Mol. Biol. Evol. 28, 2403–2417. 10.1093/molbev/msr065 21415026

[B49] Hu Y. Shin D.-J. Pan H. Lin Z. Dreyfuss J. M. Camargo F. D.. (2017). YAP suppresses gluconeogenic gene expression through PGC1α. Hepatology 66, 2029–2041. 10.1002/hep.29373 28714135 PMC6082140

[B50] Huang J. Wu S. Barrera J. Matthews K. Pan D. (2005). The Hippo signaling pathway coordinately regulates cell proliferation and apoptosis by inactivating Yorkie, the Drosophila Homolog of YAP. Cell 122, 421–434. 10.1016/j.cell.2005.06.007 16096061

[B51] Jeong H. Bae S. An S. Y. Byun M. R. Hwang J. H. Yaffe M. B.. (2010). TAZ as a novel enhancer of MyoD-mediated myogenic differentiation. FASEB J. 24, 3310–3320. 10.1096/fj.09-151324 20466877

[B52] Judson R. N. Tremblay A. M. Knopp P. White R. B. Urcia R. De Bari C.. (2012). The Hippo pathway member Yap plays a key role in influencing fate decisions in muscle satellite cells. J. Cell Sci. 125(Pt 24), 6009–6019. 10.1242/jcs.109546 23038772 PMC3585517

[B53] Kaan H. Y. K. Chan S. W. Tan S. K. J. Guo F. Lim C. J. Hong W.. (2017). Crystal structure of TAZ-TEAD complex reveals a distinct interaction mode from that of YAP-TEAD complex. Sci. Rep. 7:2035. 10.1038/s41598-017-02219-9 28515457 PMC5435683

[B54] Kim M. Kim T. Johnson R. L. Lim D. S. (2015). Transcriptional co-repressor function of the hippo pathway transducers YAP and TAZ. Cell Rep. 11, 270–282. 10.1016/j.celrep.2015.03.015 25843714

[B55] Koontz L. M. Liu-Chittenden Y. Yin F. Zheng Y. Yu J. Huang B.. (2013). The Hippo effector Yorkie controls normal tissue growth by antagonizing scalloped-mediated default repression. Dev. Cell 25, 388–401. 10.1016/j.devcel.2013.04.021 23725764 PMC3705890

[B56] Lai Z. C. Wei X. Shimizu T. Ramos E. Rohrbaugh M. Nikolaidis N.. (2005). Control of cell proliferation and apoptosis by mob as tumor suppressor, mats. Cell 120, 675–685. 10.1016/j.cell.2004.12.036 15766530

[B57] Lapi E. Di Agostino S. Donzelli S. Gal H. Domany E. Rechavi G.. (2008). PML, YAP, and p73 are components of a proapoptotic autoregulatory feedback loop. Mol. Cell 32, 803–814. 10.1016/j.molcel.2008.11.019 19111660

[B58] Lei Q. Y. Zhang H. Zhao B. Zha Z. Y. Bai F. Pei X. H.. (2008). TAZ promotes cell proliferation and epithelial-mesenchymal transition and is inhibited by the hippo pathway. Mol. Cell. Biol. 28, 2426–2436. 10.1128/MCB.01874-07 18227151 PMC2268418

[B59] Li H. Huang Z. Gao M. Huang N. Luo Z. Shen H.. (2016). Inhibition of YAP suppresses CML cell proliferation and enhances efficacy of imatinib *in vitro* and *in vivo*. J. Exp. Clin. Cancer Res. 35, 134. 10.1186/s13046-016-0414-z 27599610 PMC5012077

[B60] Li S. Cho Y. S. Yue T. Ip Y. T. Jiang J. (2015). Overlapping functions of the MAP4K family kinases Hppy and Msn in Hippo signaling. Cell Discov 1:15038. 10.1038/celldisc.2015.38 27462435 PMC4860773

[B61] Li Z. Zhao B. Wang P. Chen F. Dong Z. Yang H.. (2010). Structural insights into the YAP and TEAD complex. Genes Dev. 24, 235–240. 10.1101/gad.1865810 20123905 PMC2811825

[B62] Lian I. Kim J. Okazawa H. Zhao J. Zhao B. Yu J.. (2010). The role of YAP transcription coactivator in regulating stem cell self-renewal and differentiation. Genes Dev. 24, 1106–1118. 10.1101/gad.1903310 20516196 PMC2878649

[B63] Liang N. Zhang C. Dill P. Panasyuk G. Pion D. Koka V.. (2014). Regulation of YAP by mTOR and autophagy reveals a therapeutic target of tuberous sclerosis complex. J. Exp. Med. 211, 2249–2263. 10.1084/jem.20140341 25288394 PMC4203941

[B64] Lin L. Sabnis A. J. Chan E. Olivas V. Cade L. Pazarentzos E.. (2015). The Hippo effector YAP promotes resistance to RAF- and MEK-targeted cancer therapies. Nat. Genet. 47, 250–256. 10.1038/ng.3218 25665005 PMC4930244

[B65] Liu C. Y. Zha Z. Y. Zhou X. Zhang H. Huang W. Zhao D.. (2010). The hippo tumor pathway promotes TAZ degradation by phosphorylating a phosphodegron and recruiting the SCF^β−TrCP^ E3 ligase. J. Biol. Chem. 285, 37159–37169. 10.1074/jbc.M110.152942 20858893 PMC2988322

[B66] Liu F. Lagares D. Choi K. M. Stopfer L. Marinkovic A. Vrbanac V.. (2015). Mechanosignaling through YAP and TAZ drives fibroblast activation and fibrosis. Am. J. Physiol. Lung Cell. Mol. Physiol. 308, L344–357. 10.1152/ajplung.00300.2014 25502501 PMC4329470

[B67] Liu-Chittenden Y. Huang B. Shim J. S. Chen Q. Lee S. J. Anders R. A.. (2012). Genetic and pharmacological disruption of the TEAD-YAP complex suppresses the oncogenic activity of YAP. Genes Dev. 26, 1300–1305. 10.1101/gad.192856.112 22677547 PMC3387657

[B68] Masuda T. Ishitani T. (2017). Context-dependent regulation of the β-catenin transcriptional complex supports diverse functions of Wnt/β-catenin signaling. J. Biochem. 161, 9–17. 10.1093/jb/mvw072 28013224

[B69] Matsui Y. Nakano N. Shao D. Gao S. Luo W. Hong C.. (2008). Lats2 is a negative regulator of myocyte size in the heart. Circ. Res. 103, 1309–1318. 10.1161/CIRCRESAHA.108.180042 18927464 PMC2775813

[B70] Meng Z. Moroishi T. Guan K. L. (2016). Mechanisms of Hippo pathway regulation. Genes Dev. 30, 1–17. 10.1101/gad.274027.115 26728553 PMC4701972

[B71] Meng Z. Moroishi T. Mottier-Pavie V. Plouffe S. W. Hansen C. G. Hong A. W.. (2015). MAP4K family kinases act in parallel to MST1/2 to activate LATS1/2 in the Hippo pathway. Nat. Commun. 6:8357. 10.1038/ncomms9357 26437443 PMC4600732

[B72] Miller E. Yang J. DeRan M. Wu C. Su A. I. Bonamy G. M.. (2012). Identification of serum-derived sphingosine-1-phosphate as a small molecule regulator of YAP. Chem. Biol. 19, 955–962. 10.1016/j.chembiol.2012.07.005 22884261

[B73] Mo J. S. Meng Z. Kim Y. C. Park H. W. Hansen C. G. Kim S.. (2015). Cellular energy stress induces AMPK-mediated regulation of YAP and the Hippo pathway. Nat. Cell Biol. 17, 500–510. 10.1038/ncb3111 25751140 PMC4380774

[B74] Mohamed A. Sun C. De Mello V. Selfe J. Missiaglia E. Shipley J.. (2016). The Hippo effector TAZ (WWTR1) transforms myoblasts and TAZ abundance is associated with reduced survival in embryonal rhabdomyosarcoma. J. Pathol. 240, 3–14. 10.1002/path.4745 27184927 PMC4995731

[B75] Morikawa Y. Heallen T. Leach J. Xiao Y. Martin J. F. (2017). Dystrophin-glycoprotein complex sequesters Yap to inhibit cardiomyocyte proliferation. Nature 547, 227–231. 10.1038/nature22979 28581498 PMC5528853

[B76] Morin-Kensicki E. M. Boone B. N. Howell M. Stonebraker J. R. Teed J. Alb J. G.. (2006). Defects in yolk sac vasculogenesis, chorioallantoic fusion, and embryonic axis elongation in mice with targeted disruption of Yap65. Mol. Cell. Biol. 26, 77–87. 10.1128/MCB.26.1.77-87.2006 16354681 PMC1317614

[B77] Moroishi T. Park H. W. Qin B. Chen Q. Meng Z. Plouffe S. W.. (2015). A YAP/TAZ-induced feedback mechanism regulates Hippo pathway homeostasis. Genes Dev. 29, 1271–1284. 10.1101/gad.262816.115 26109050 PMC4495398

[B78] Murakami M. Nakagawa M. Olson E. N. Nakagawa O. (2005). A WW domain protein TAZ is a critical coactivator for TBX5, a transcription factor implicated in Holt-Oram syndrome. Proc. Natl. Acad. Sci. U.S.A. 102, 18034–18039. 10.1073/pnas.0509109102 16332960 PMC1312418

[B79] Nagaraj R. Gururaja-Rao S. Jones K. T. Slattery M. Negre N. Braas D.. (2012). Control of mitochondrial structure and function by the Yorkie/YAP oncogenic pathway. Genes Dev. 26, 2027–2037. 10.1101/gad.183061.111 22925885 PMC3444729

[B80] Nakajima H. Yamamoto K. Agarwala S. Terai K. Fukui H. Fukuhara S.. (2017). Flow-dependent endothelial YAP regulation contributes to vessel maintenance. Dev. Cell 40, 523–536 e526. 10.1016/j.devcel.2017.02.019 28350986

[B81] Oh H. Slattery M. Ma L. Crofts A. White K. P. Mann R. S.. (2013). Genome-wide association of Yorkie with chromatin and chromatin-remodeling complexes. Cell Rep. 3, 309–318. 10.1016/j.celrep.2013.01.008 23395637 PMC3633442

[B82] Oudhoff M. J. Freeman S. A. Couzens A. L. Antignano F. Kuznetsova E. Min P. H.. (2013). Control of the hippo pathway by Set7-dependent methylation of Yap. Dev. Cell 26, 188–194. 10.1016/j.devcel.2013.05.025 23850191

[B83] Pantalacci S. Tapon N. Leopold P. (2003). The Salvador partner Hippo promotes apoptosis and cell-cycle exit in Drosophila. Nat. Cell Biol. 5, 921–927. 10.1038/ncb1051 14502295

[B84] Paramasivam M. Sarkeshik A. Yates J. R. III. Fernandes M. J. McCollum D. (2011). Angiomotin family proteins are novel activators of the LATS2 kinase tumor suppressor. Mol. Biol. Cell 22, 3725–3733. 10.1091/mbc.E11-04-0300 21832154 PMC3183025

[B85] Park G. H. Jeong H. Jeong M. G. Jang E. J. Bae M. A. Lee Y. L.. (2014). Novel TAZ modulators enhance myogenic differentiation and muscle regeneration. Br. J. Pharmacol. 171, 4051–4061. 10.1111/bph.12755 24821191 PMC4243978

[B86] Park G. S. Oh H. Kim M. Kim T. Johnson R. L. Irvine K. D.. (2016). An evolutionarily conserved negative feedback mechanism in the Hippo pathway reflects functional difference between LATS1 and LATS2. Oncotarget 7, 24063–24075. 10.18632/oncotarget.8211 27006470 PMC5029684

[B87] Park H. W. Kim Y. C. Yu B. Moroishi T. Mo J. S. Plouffe S. W.. (2015). Alternative Wnt Signaling Activates YAP/TAZ. Cell 162, 780–794. 10.1016/j.cell.2015.07.013 26276632 PMC4538707

[B88] Park Y. Y. Sohn B. H. Johnson R. L. Kang M. H. Kim S. B. Shim J. J.. (2016). Yes-associated protein 1 and transcriptional coactivator with PDZ-binding motif activate the mammalian target of rapamycin complex 1 pathway by regulating amino acid transporters in hepatocellular carcinoma. Hepatology 63, 159–172. 10.1002/hep.28223 26389641 PMC4881866

[B89] Patel S. H. Camargo F. D. Yimlamai D. (2017). Hippo signaling in the liver regulates organ size, cell fate, and carcinogenesis. Gastroenterology 152, 533–545. 10.1053/j.gastro.2016.10.047 28003097 PMC5285449

[B90] Pegoraro S. Ros G. Ciani Y. Sgarra R. Piazza S. Manfioletti G. (2015). A novel HMGA1-CCNE2-YAP axis regulates breast cancer aggressiveness. Oncotarget 6, 19087–19101. 10.18632/oncotarget.4236 26265440 PMC4662477

[B91] Plouffe S. W. Meng Z. Lin K. C. Lin B. Hong A. W. Chun J. V.. (2016). Characterization of Hippo pathway components by gene inactivation. Mol. Cell 64, 993–1008. 10.1016/j.molcel.2016.10.034 27912098 PMC5137798

[B92] Poon C. L. Lin J. I. Zhang X. Harvey K. F. (2011). The sterile 20-like kinase Tao-1 controls tissue growth by regulating the Salvador-Warts-Hippo pathway. Dev. Cell 21, 896–906. 10.1016/j.devcel.2011.09.012 22075148

[B93] Porazinski S. Wang H. Asaoka Y. Behrndt M. Miyamoto T. Morita H.. (2015). YAP is essential for tissue tension to ensure vertebrate 3D body shape. Nature 521, 217–221. 10.1038/nature14215 25778702 PMC4720436

[B94] Praskova M. Khoklatchev A. Ortiz-Vega S. Avruch J. (2004). Regulation of the MST1 kinase by autophosphorylation, by the growth inhibitory proteins, RASSF1 and NORE1, and by Ras. Biochem. J. 381(Pt 2), 453–462. 10.1042/BJ20040025 15109305 PMC1133852

[B95] Praskova M. Xia F. Avruch J. (2008). MOBKL1A/MOBKL1B phosphorylation by MST1 and MST2 inhibits cell proliferation. Curr. Biol. 18, 311–321. 10.1016/j.cub.2008.02.006 18328708 PMC4682548

[B96] Purvis J. E. Lahav G. (2013). Encoding and decoding cellular information through signaling dynamics. Cell 152, 945–956. 10.1016/j.cell.2013.02.005 23452846 PMC3707615

[B97] Reddy B. V. Irvine K. D. (2013). Regulation of Hippo signaling by EGFR-MAPK signaling through Ajuba family proteins. Dev. Cell 24, 459–471. 10.1016/j.devcel.2013.01.020 23484853 PMC3624988

[B98] Ribeiro P. S. Josue F. Wepf A. Wehr M. C. Rinner O. Kelly G.. (2010). Combined functional genomic and proteomic approaches identify a PP2A complex as a negative regulator of Hippo signaling. Mol. Cell 39, 521–534. 10.1016/j.molcel.2010.08.002 20797625

[B99] Richardson H. E. Portela M. (2017). Tissue growth and tumorigenesis in Drosophila: cell polarity and the Hippo pathway. Curr. Opin. Cell Biol. 48, 1–9. 10.1016/j.ceb.2017.03.006 28364663

[B100] Rosenbluh J. Nijhawan D. Cox A. G. Li X. Neal J. T. Schafer E. J.. (2012). β-Catenin-driven cancers require a YAP1 transcriptional complex for survival and tumorigenesis. Cell 151, 1457–1473. 10.1016/j.cell.2012.11.026 23245941 PMC3530160

[B101] Sansores-Garcia L. Bossuyt W. Wada K. Yonemura S. Tao C. Sasaki H.. (2011). Modulating F-actin organization induces organ growth by affecting the Hippo pathway. EMBO J. 30, 2325–2335. 10.1038/emboj.2011.157 21556047 PMC3116287

[B102] Sasaki H. (2017). Roles and regulations of Hippo signaling during preimplantation mouse development. Dev. Growth Differ. 59, 12–20. 10.1111/dgd.12335 28035666

[B103] Schlegelmilch K. Mohseni M. Kirak O. Pruszak J. Rodriguez J. R. Zhou D.. (2011). Yap1 acts downstream of alpha-catenin to control epidermal proliferation. Cell 144, 782–795. 10.1016/j.cell.2011.02.031 21376238 PMC3237196

[B104] Shao D. Zhai P. Del Re D. P. Sciarretta S. Yabuta N. Nojima H.. (2014). A functional interaction between Hippo-YAP signalling and FoxO1 mediates the oxidative stress response. Nat. Commun. 5:3315. 10.1038/ncomms4315 24525530 PMC3962829

[B105] Sorrentino G. Ruggeri N. Specchia V. Cordenonsi M. Mano M. Dupont S.. (2014). Metabolic control of YAP and TAZ by the mevalonate pathway. Nat. Cell Biol. 16, 357–366. 10.1038/ncb2936 24658687

[B106] Sorrentino G. Ruggeri N. Zannini A. Ingallina E. Bertolio R. Marotta C.. (2017). Glucocorticoid receptor signalling activates YAP in breast cancer. Nat. Commun. 8:14073. 10.1038/ncomms14073 28102225 PMC5253666

[B107] Strano S. Munarriz E. Rossi M. Castagnoli L. Shaul Y. Sacchi A.. (2001). Physical interaction with Yes-associated protein enhances p73 transcriptional activity. J. Biol. Chem. 276, 15164–15173. 10.1074/jbc.M010484200 11278685

[B108] Strassburger K. Tiebe M. Pinna F. Breuhahn K. Teleman A. A. (2012). Insulin/IGF signaling drives cell proliferation in part via Yorkie/YAP. Dev. Biol. 367, 187–196. 10.1016/j.ydbio.2012.05.008 22609549

[B109] Sun C. De Mello V. Mohamed A. Ortuste Quiroga H. P. Garcia-Munoz A. Al Bloshi A.. (2017). Common and distinctive functions of the Hippo effectors Taz and Yap in skeletal muscle stem cell function. Stem Cells. 35, 1958–1972. 10.1002/stem.2652 28589555 PMC5575518

[B110] Tamm C. Bower N. Anneren C. (2011). Regulation of mouse embryonic stem cell self-renewal by a Yes-YAP-TEAD2 signaling pathway downstream of LIF. J. Cell Sci. 124(Pt 7), 1136–1144. 10.1242/jcs.075796 21385842

[B111] Tang F. Gill J. Ficht X. Barthlott T. Cornils H. Schmitz-Rohmer D.. (2015). The kinases NDR1/2 act downstream of the Hippo homolog MST1 to mediate both egress of thymocytes from the thymus and lymphocyte motility. Sci. Signal. 8:ra100. 10.1126/scisignal.aab2425 26443704

[B112] Taniguchi K. Wu L. W. Grivennikov S. I. de Jong P. R. Lian I. Yu F. X.. (2015). A gp130-Src-YAP module links inflammation to epithelial regeneration. Nature 519, 57–62. 10.1038/nature14228 25731159 PMC4447318

[B113] Tapon N. Harvey K. F. Bell D. W. Wahrer D. C. Schiripo T. A. Haber D.. (2002). Salvador promotes both cell cycle exit and apoptosis in Drosophila and is mutated in human cancer cell lines. Cell 110, 467–478. 10.1016/S0092-8674(02)00824-3 12202036

[B114] Totaro A. Castellan M. Battilana G. Zanconato F. Azzolin L. Giulitti S.. (2017). YAP/TAZ link cell mechanics to Notch signalling to control epidermal stem cell fate. Nat. Commun. 8:15206. 10.1038/ncomms15206 28513598 PMC5442321

[B115] Tremblay A. M. Missiaglia E. Galli G. G. Hettmer S. Urcia R. Carrara M.. (2014). The Hippo transducer YAP1 transforms activated satellite cells and is a potent effector of embryonal rhabdomyosarcoma formation. Cancer Cell 26, 273–287. 10.1016/j.ccr.2014.05.029 25087979

[B116] Udan R. S. Kango-Singh M. Nolo R. Tao C. Halder G. (2003). Hippo promotes proliferation arrest and apoptosis in the Salvador/Warts pathway. Nat. Cell Biol. 5, 914–920. 10.1038/ncb1050 14502294

[B117] Varelas X. Miller B. W. Sopko R. Song S. Gregorieff A. Fellouse F. A.. (2010a). The Hippo pathway regulates Wnt/beta-catenin signaling. Dev. Cell 18, 579–591. 10.1016/j.devcel.2010.03.007 20412773

[B118] Varelas X. Samavarchi-Tehrani P. Narimatsu M. Weiss A. Cockburn K. Larsen B. G.. (2010b). The Crumbs complex couples cell density sensing to Hippo-dependent control of the TGF-beta-SMAD pathway. Dev. Cell 19, 831–844. 10.1016/j.devcel.2010.11.012 21145499

[B119] Vassilev A. Kaneko K. J. Shu H. Zhao Y. DePamphilis M. L. (2001). TEAD/TEF transcription factors utilize the activation domain of YAP65, a Src/Yes-associated protein localized in the cytoplasm. Genes Dev. 15, 1229–1241. 10.1101/gad.888601 11358867 PMC313800

[B120] von Eyss B. Jaenicke L. A. Kortlever R. M. Royla N. Wiese K. E. Letschert S.. (2015). A MYC-Driven Change in Mitochondrial Dynamics Limits YAP/TAZ function in mammary epithelial cells and breast cancer. Cancer Cell 28, 743–757. 10.1016/j.ccell.2015.10.013 26678338

[B121] Wada K. Itoga K. Okano T. Yonemura S. Sasaki H. (2011). Hippo pathway regulation by cell morphology and stress fibers. Development 138, 3907–3914. 10.1242/dev.070987 21831922

[B122] Wang K. C. Yeh Y. T. Nguyen P. Limqueco E. Lopez J. Thorossian S.. (2016). Flow-dependent YAP/TAZ activities regulate endothelial phenotypes and atherosclerosis. Proc. Natl. Acad. Sci. U.S.A. 113, 11525–11530. 10.1073/pnas.1613121113 27671657 PMC5068257

[B123] Wang L. Luo J.-Y. Li B. Tian X. Y. Chen L. J. Huang Y. (2016). Integrin-YAP/TAZ-JNK cascade mediates atheroprotective effect of unidirectional shear flow. Nature 540, 579–582. 10.1038/nature20602 27926730

[B124] Wang W. Huang J. Chen J. (2011). Angiomotin-like proteins associate with and negatively regulate YAP1. J. Biol. Chem. 286, 4364–4370. 10.1074/jbc.C110.205401 21187284 PMC3039387

[B125] Wang W. Xiao Z. D. Li X. Aziz K. E. Gan B. Johnson R. L.. (2015). AMPK modulates Hippo pathway activity to regulate energy homeostasis. Nat. Cell Biol. 17, 490–499. 10.1038/ncb3113 25751139 PMC4380807

[B126] Wang Z. Wu Y. Wang H. Zhang Y. Mei L. Fang X.. (2014). Interplay of mevalonate and Hippo pathways regulates RHAMM transcription via YAP to modulate breast cancer cell motility. Proc. Natl. Acad. Sci. U.S.A. 111, E89–E98. 10.1073/pnas.1319190110 24367099 PMC3890879

[B127] Watt K. I. Judson R. Medlow P. Reid K. Kurth T. B. Burniston J. G.. (2010). Yap is a novel regulator of C2C12 myogenesis. Biochem. Biophys. Res. Commun. 393, 619–624. 10.1016/j.bbrc.2010.02.034 20153295

[B128] Watt K. I. Turner B. J. Hagg A. Zhang X. Davey J. R. Qian H.. (2015). The Hippo pathway effector YAP is a critical regulator of skeletal muscle fibre size. Nat. Commun. 6:6048. 10.1038/ncomms7048 25581281

[B129] Wu H. Wei L. Fan F. Ji S. Zhang S. Geng J.. (2015). Integration of Hippo signalling and the unfolded protein response to restrain liver overgrowth and tumorigenesis. Nat. Commun. 6:6239. 10.1038/ncomms7239 25695629

[B130] Wu S. Huang J. Dong J. Pan D. (2003). hippo encodes a Ste-20 family protein kinase that restricts cell proliferation and promotes apoptosis in conjunction with salvador and warts. Cell 114, 445–456. 10.1016/S0092-8674(03)00549-X 12941273

[B131] Wu S. Liu Y. Zheng Y. Dong J. Pan D. (2008). The TEAD/TEF family protein Scalloped mediates transcriptional output of the Hippo growth-regulatory pathway. Dev. Cell 14, 388–398. 10.1016/j.devcel.2008.01.007 18258486

[B132] Xin M. Kim Y. Sutherland L. B. Murakami M. Qi X. McAnally J.. (2013). Hippo pathway effector Yap promotes cardiac regeneration. Proc. Natl. Acad. Sci. U.S.A. 110, 13839–13844. 10.1073/pnas.1313192110 23918388 PMC3752208

[B133] Yan L. Cai Q. Xu Y. (2014). Hypoxic conditions differentially regulate TAZ and YAP in cancer cells. Arch. Biochem. Biophys. 562, 31–36. 10.1016/j.abb.2014.07.024 25078107 PMC4197065

[B134] Yang S. Zhang L. Liu M. Chong R. Ding S. J. Chen Y.. (2013). CDK1 phosphorylation of YAP promotes mitotic defects and cell motility and is essential for neoplastic transformation. Cancer Res. 73, 6722–6733. 10.1158/0008-5472.CAN-13-2049 24101154 PMC3861241

[B135] Yu F. X. Zhao B. Panupinthu N. Jewell J. L. Lian I. Wang L. H.. (2012). Regulation of the Hippo-YAP pathway by G-protein-coupled receptor signaling. Cell 150, 780–791. 10.1016/j.cell.2012.06.037 22863277 PMC3433174

[B136] Zaidi S. K. Sullivan A. J. Medina R. Ito Y. van Wijnen A. J. Stein J. L.. (2004). Tyrosine phosphorylation controls Runx2-mediated subnuclear targeting of YAP to repress transcription. EMBO J. 23, 790–799. 10.1038/sj.emboj.7600073 14765127 PMC380991

[B137] Zanconato F. Forcato M. Battilana G. Azzolin L. Quaranta E. Bodega B.. (2015). Genome-wide association between YAP/TAZ/TEAD and AP-1 at enhancers drives oncogenic growth. Nat. Cell Biol. 17, 1218–1227. 10.1038/ncb3216 26258633 PMC6186417

[B138] Zeng Y. Stauffer S. Zhou J. Chen X. Chen Y. Dong J. (2017). Cyclin-dependent kinase 1 (CDK1)-mediated mitotic phosphorylation of the transcriptional co-repressor Vgll4 inhibits its tumor-suppressing activity. J. Biol. Chem. 292, 15028–15038. 10.1074/jbc.M117.796284 28739871 PMC5592678

[B139] Zhang L. Ren F. Zhang Q. Chen Y. Wang B. Jiang J. (2008). The TEAD/TEF family of transcription factor Scalloped mediates Hippo signaling in organ size control. Dev. Cell 14, 377–387. 10.1016/j.devcel.2008.01.006 18258485 PMC2292673

[B140] Zhang L. Tang F. Terracciano L. Hynx D. Kohler R. Bichet S.. (2015). NDR functions as a physiological YAP1 kinase in the intestinal epithelium. Curr. Biol. 25, 296–305. 10.1016/j.cub.2014.11.054 25601544 PMC4426889

[B141] Zhang Y. Del Re D. P. (2017). A growing role for the Hippo signaling pathway in the heart. J. Mol. Med. 95, 465–472. 10.1007/s00109-017-1525-5 28280861 PMC5404975

[B142] Zhao B. Kim J. Ye X. Lai Z. C. Guan K. L. (2009). Both TEAD-binding and WW domains are required for the growth stimulation and oncogenic transformation activity of yes-associated protein. Cancer Res. 69, 1089–1098. 10.1158/0008-5472.CAN-08-2997 19141641

[B143] Zhao B. Li L. Lu Q. Wang L. H. Liu C. Y. Lei Q.. (2011). Angiomotin is a novel Hippo pathway component that inhibits YAP oncoprotein. Genes Dev. 25, 51–63. 10.1101/gad.2000111 21205866 PMC3012936

[B144] Zhao B. Li L. Tumaneng K. Wang C. Y. Guan K. L. (2010). A coordinated phosphorylation by Lats and CK1 regulates YAP stability through SCF^β−TRCP^. Genes Dev. 24, 72–85. 10.1101/gad.1843810 20048001 PMC2802193

[B145] Zhao B. Wei X. Li W. Udan R. S. Yang Q. Kim J.. (2007). Inactivation of YAP oncoprotein by the Hippo pathway is involved in cell contact inhibition and tissue growth control. Genes Dev. 21, 2747–2761. 10.1101/gad.1602907 17974916 PMC2045129

[B146] Zhao B. Ye X. Yu J. Li L. Li W. Li S.. (2008). TEAD mediates YAP-dependent gene induction and growth control. Genes Dev. 22, 1962–1971. 10.1101/gad.1664408 18579750 PMC2492741

[B147] Zhao X. Wang X. Fang L. Lan C. Zheng X. Wang Y.. (2017). A combinatorial strategy using YAP and pan-RAF inhibitors for treating KRAS-mutant pancreatic cancer. Cancer Lett. 402, 61–70. 10.1016/j.canlet.2017.05.015 28576749

[B148] Zhao Y. Khanal P. Savage P. She Y. M. Cyr T. D. Yang X. (2014). YAP-induced resistance of cancer cells to antitubulin drugs is modulated by a Hippo-independent pathway. Cancer Res. 74, 4493–4503. 10.1158/0008-5472.CAN-13-2712 24812269

[B149] Zheng Y. Wang W. Liu B. Deng H. Uster E. Pan D. (2015). Identification of Happyhour/MAP4K as Alternative Hpo/Mst-like Kinases in the Hippo Kinase Cascade. Dev. Cell 34, 642–655. 10.1016/j.devcel.2015.08.014 26364751 PMC4589524

[B150] Zhou W. Liotta L. A. Petricoin E. F. (2017). The warburg effect and mass spectrometry-based proteomic analysis. Cancer Genomics Proteomics 14, 211–218. 10.21873/cgp.20032 28647695 PMC5572299

[B151] Zhou X. Wang S. Wang Z. Feng X. Liu P. Lv X. B.. (2015). Estrogen regulates Hippo signaling via GPER in breast cancer. J. Clin. Invest. 125, 2123–2135. 10.1172/JCI79573 25893606 PMC4463207

